# Evaluation and Optimization of Prediction Models for Crop Yield in Plant Factory

**DOI:** 10.3390/plants14142140

**Published:** 2025-07-10

**Authors:** Yaoqi Peng, Yudong Zheng, Zengwei Zheng, Yong He

**Affiliations:** 1 College of Biosystems Engineering and Food Science, Zhejiang University, Hangzhou 310058, China; pengyaoqi@yeah.net; 2 School of Computer and Computing Science, Hangzhou City University, Hangzhou 310015, China; 3 Zhejiang Provincial Engineering Research Center for Intelligent Plant Factory, Hangzhou 310015, China; 4 Key Laboratory of Crop Drought Resistance Research of Hebei Province, Institute of Dryland Farming, Hebei Academy of Agriculture and Forestry Sciences, Hengshui 053000, China; 15612771262@163.com

**Keywords:** crop canopy image, plant factory, crop yield prediction, Wide Neural Network

## Abstract

This study focuses on enhancing crop yield prediction in plant factory environments through precise crop canopy image capture and background interference removal. This method achieves highly accurate recognition of the crop canopy projection area (CCPA), with a coefficient of determination (R^2^) of 0.98. A spatial resolution of 0.078 mm/pixel was derived by referencing a scale ruler and processing pixel counts, eliminating outliers in the data. Image post-processing focused on extracting the canopy boundary and calculating the crop canopy area. By incorporating crop yield data, a comparative analysis of 28 prediction models was performed, assessing performance metrics such as MSE, RMSE, MAE, MAPE, R^2^, prediction speed, training time, and model size. Among them, the Wide Neural Network model emerged as the most optimal. It demonstrated remarkable predictive accuracy with an R^2^ of 0.95, RMSE of 27.15 g, and MAPE of 11.74%. Furthermore, the model achieved a high prediction speed of 60,234.9 observations per second, and its compact size of 7039 bytes makes it suitable for efficient, real-time deployment in practical applications. This model offers substantial support for managing crop growth, providing a solid foundation for refining cultivation processes and enhancing crop yields.

## 1. Introduction

With the continuous growth of the global population and the rapid pace of urbanization, food security and sustainable development have become pressing concerns [1,2,3]. Traditional agricultural production methods are encountering numerous challenges, including limited land resources, climate change, and water shortages, which place unprecedented stress on agricultural sustainability [4,5]. Additionally, the overuse of pesticides and fertilizers has caused non-point source pollution in agricultural fields, leading to significant environmental contamination and ecological degradation, thus jeopardizing ecosystem stability and human health [6,7,8]. Consequently, it is imperative to explore and implement novel agricultural production techniques to achieve sustainable agricultural development.

As an innovative model of modern agriculture, plant factories leverage advanced facility agriculture technologies to create a controlled environment that enables precise regulation of the entire plant growth process [9,10,11]. This integrated system can dynamically adjust factors such as temperature, humidity, light intensity, and CO_2_ concentration, optimizing the growing environment to enhance both production efficiency and crop quality [12,13]. Plant factories not only effectively conserve land, water, and energy resources, but also significantly reduce the use of chemical pesticides and fertilizers, thereby lessening the environmental burden and safeguarding ecosystems [14,15,16]. These advantages position plant factories as a viable solution to the challenges of resource scarcity and environmental pollution in current agricultural production, providing strong support for the green and sustainable development of agriculture [17,18]. Crop yield is a critical reference indicator for environmental feedback regulation in plant factories [19,20]. Achieving rapid yield prediction under plant factory conditions has become a focal point of research for many scholars.

There are many ways to realize crop yield prediction. Iniyan et al. utilized an array of sensors to gather environmental parameter data from multiple regions, including temperature, precipitation, humidity, soil type, crop species, seasonal variation, and field area, which served as the basis for precise crop yield forecasting [21]. Qiao et al. integrated multispectral and multitemporal remote sensing imagery data, leveraging the multidimensional information across spatial, spectral, and temporal dimensions. They developed a comprehensive spatiotemporal–spectral feature extraction framework aimed at addressing the imbalance in crop yield label distribution, thereby optimizing the accuracy of crop yield prediction [22]. Gene-Mola et al. adopted a combination of air-assisted spraying technology with forced airflows and multi-view sensors to thoroughly capture the three-dimensional structural features of apples. By leveraging LiDAR technology for 3D plant modeling, they effectively improved the precision of crop yield forecasting [23]. Existing studies often rely on the deployment of numerous environmental sensors or costly spectral equipment. While these high-end devices can provide high-precision data, their exorbitant acquisition costs and complex setup processes impose significant limitations on practical applications, making them both time-consuming and resource-intensive. Therefore, designing a low-cost, user-friendly portable data acquisition method for efficient crop characterization becomes particularly crucial.

The accuracy of crop yield prediction largely depends on the modeling methods employed. Chen et al. successfully calibrated the Decision Support System for Agrotechnology Transfer (DSSAT) model using leaf area index values and plant nitrogen accumulation (PNA) values generated from spectral indices, they were able to more accurately reflect the growth status and nutritional requirements of crops [24]. Zare et al. employed the Particle Filter (PF) algorithm for intra-seasonal crop yield prediction, effectively assimilating leaf area index data into three individual crop models (CERES, GECROS, and SPASS), as well as their multi-model ensemble (MME), to address the common issue of missing data in large-scale agricultural monitoring [25]. The temporal convolutional network (TCN) proposed by Mohan et al. incorporates a specially designed dilated convolution module, enabling the prediction of rice crop yield based on vegetation indices and climatic factors [26]. Current crop yield prediction studies predominantly focus on outdoor field conditions, while research on yield prediction in controlled environments, such as plant factories, remains limited. The applicability of existing yield prediction models in these highly controlled environments may not be straightforward, and their adaptability and effectiveness need to be further assessed.

Considering the cost-effectiveness of data collection equipment and the adaptability of predictive models, this study proposes the use of smartphones as low-cost data acquisition terminals. The crop canopy images will be captured through a close-up, vertical top-down perspective. For the extraction of crop canopy extent, morphological image processing techniques are integrated with an active contour model, aiming to achieve efficient recognition and precise modeling of the crop canopy’s outline. In the post-processing phase, the crop canopy area is further computed based on the extracted canopy outline. Using this data, along with crop yield information, various prediction models are introduced to forecast crop yield. The goal is to optimize model performance and select the most suitable prediction method, thereby enhancing the model’s adaptability and prediction accuracy in practical applications. Ultimately, through systematic comparison and analysis, the most advantageous prediction method will be selected, providing both theoretical and practical guidance for future research on precision agriculture and crop yield prediction.

## 2. Materials and Methods

### 2.1. Crop Growth Area

**Figure 1:**
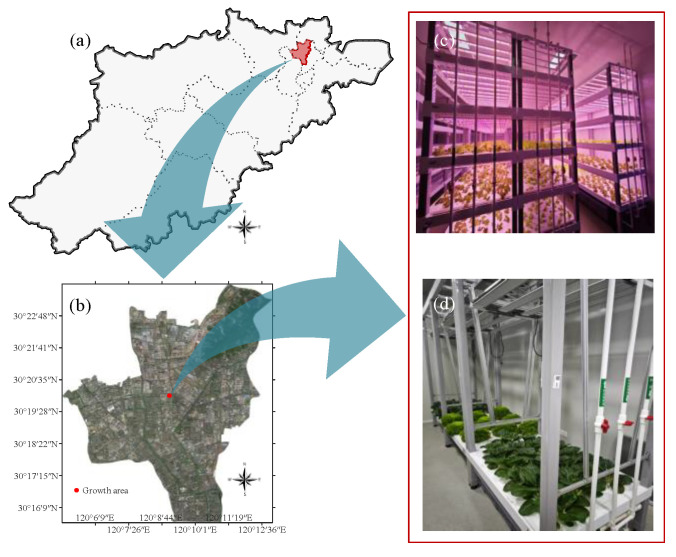
Crop growth area. (**a**) Administrative divisions of Hangzhou; (**b**) satellite map of Gongshu District; (**c**) seedling room; (**d**) cultivation room.

This study was conducted at the Zhejiang Provincial Engineering Research Center for Intelligent Plant Factory (120°9′25″ N, 30°19′55″ E), focusing on crop cultivation (Figure 1). The facility consists of two primary sections: the seedling room and the cultivation room. The seedling room is dedicated to seed germination and early-stage seedling growth, typically featuring multi-tiered planting racks to optimize space utilization and facilitate monitoring. The cultivation room serves as the primary environment for crop development from seedlings to mature plants, with its spatial configuration tailored to the specific needs and growth habits of the crops.

**Figure 2:**
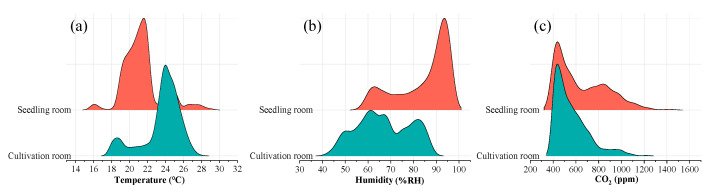
Environmental data of planting area. (**a**) Temperature index; (**b**) humidity index; (**c**) CO_2_ concentration index.

The growth cycle of crops from seedling transplanting to mature harvest lasts approximately 30 days. Based on environmental data collected from room sensors between 12 April 2024 and 24 May 2024 (Figure 2), it is observed that the temperature in the seedling room primarily ranges between 18 °C and 23 °C, while the temperature in the cultivation room falls between 22 °C and 27 °C. This temperature variation is attributed to the proximity of the cultivation room to the material passage, where frequent door openings result in the influx of heat, thus raising the temperature. Regarding humidity, the seedling room’s relative humidity ranges from 65% RH to 95% RH, while the cultivation room’s humidity ranges from 50% RH to 85% RH. This difference is due to the higher seedling planting density in the seedling room and the relatively enclosed environment, which limits ventilation. As for CO_2_ concentration, both rooms exhibit similar levels, with the seedling room showing a concentration between 400 ppm and 1000 ppm and the cultivation room ranging from 400 ppm to 800 ppm, indicating no significant difference between them.

### 2.2. Crop Canopy Image Acquisition

**Figure 3:**
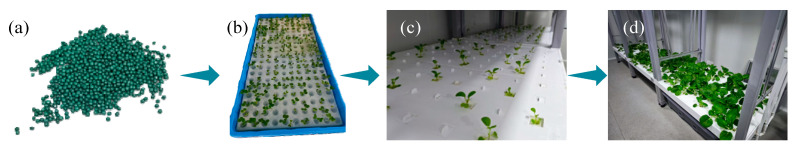
The cultivation process in plant factory production. (**a**) Cabbage seeds; (**b**) seed pre-germination; (**c**) seedling establishment; (**d**) transplanting of mature plants.

In this study, the crop grown is Black Leaf Chinese Cabbage (Brassica rapa var. chinensis), a cultivar of the Brassicaceae family [27]. It is highly regarded for its elliptical leaves, deep green and lustrous appearance, thick leaf structure, short and robust white petioles, sweetness, tenderness, low fiber content, and its high quality and excellent post-harvest storage and transportability. The cultivation process in plant factory production is divided into three phases: seed pre-germination, seedling establishment, and transplanting of mature plants (Figure 3). Each phase demands meticulous environmental parameter regulation to optimize growth conditions, ensuring the healthy development and high-quality formation of the crop.

**Figure 4:**
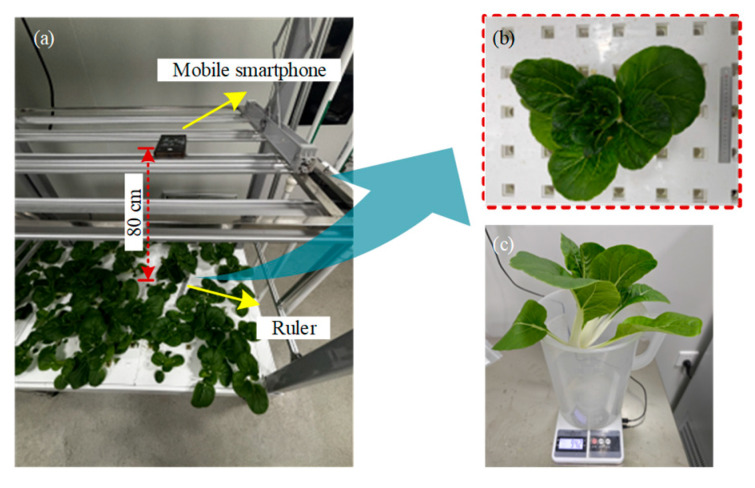
Experimental collection scene. (**a**) Crop canopy image shooting; (**b**) partial enlarged image; (**c**) plant weighing.

The experiment aims to collect canopy characteristic images of crops with different morphological forms. A mobile smartphone (Xiaomi 13 Pro) was used as the imaging device, capturing images in a vertical, top-down orientation. During the process, the camera lens was maintained at an approximate distance of 80 cm from the crop roots to ensure consistency in the imaging conditions and comparability of the data. To minimize errors that may arise due to variations in camera height relative to the crop canopy during the shooting process, a ruler was placed near the root of each crop (Figure 4).

The ruler served as a visual reference during image capture and was also used to establish a mapping relationship between the physical scale and image pixels, facilitating accurate size calibration. The calibration process effectively reduced errors caused by camera angle and positional variations, thereby enhancing the accuracy of image analysis. Following each image capture, the root section of each crop was cut and weighed, with the data recorded to support subsequent yield prediction modeling.

### 2.3. Optimization Design of Crop Yield Prediction Model

In this study, a total of 60 cabbage canopy images were collected and initially processed using color features for canopy recognition, followed by contour extraction. The collected images of crops at different growth stages, ranging from seedlings to full maturity. This strategy enables dynamic yield prediction through the continuous monitoring of the crop’s growth, circumventing the limitations of traditional methods that rely on static data and delayed predictions. This process provides an initial boundary of the cabbage canopy. To enhance the accuracy of recognition, morphological operations were employed to process the images, filling any potential internal gaps.

Morphological operations are image processing techniques that analyze and manipulate the structural information of an image based on its shape characteristics. Erosion and dilation are two fundamental operations within this domain, widely applied in tasks such as noise removal, hole filling, and object extraction. Specifically, erosion can be understood as a process of “shrinking” the target region. Essentially, it involves convolving the local region of the image with a structuring element, resulting in the contraction of the image boundaries. This operation effectively removes smaller, insignificant objects or noise within the image. The mathematical formulation of erosion is as follows:

(1) $$A⊝Β=\left\{\begin{array}{c} x,y \end{array}\right.|{\left(B\right)}_{xy}\subseteq A\}$$

where represents the erosion of *A* using structuring element *B*. It is important to note that *B* must define a specific origin. When the origin of *B* is shifted to the pixel (*x*, *y*) in image *A*, if *B* is entirely contained within the overlapping region of image *A* at (*x*, *y*), the corresponding pixel in the output image will be assigned a value of 1. Otherwise, it will be assigned a value of 0.

The primary function of the dilation operation is to expand the boundaries of the target region, incorporating background pixels that are in contact with the target region, thereby achieving an “expansion” of the target area. Specifically, the dilation operation involves convolving the image with a structuring element, causing the boundaries of the target region to expand outward. This process effectively fills any holes within the target area and removes small noise particles inside the region. The mathematical formulation of the dilation operation is as follows:

(2) $$A⊕Β=\left\{\begin{array}{c} x,y \end{array}\right.|{\left(B\right)}_{xy}\cap A\neq ⊘\}$$

where represents the dilation of image *A* using structuring element *B*. The origin of the structuring element *B* is shifted to the pixel (*x*, *y*) in the image. If the intersection between *B* and *A* at the pixel (*x*, *y*) is non-empty, the corresponding pixel in the output image will be assigned a value of 1; otherwise, it will be assigned a value of 0.

Additionally, an active contour model was applied to refine the edges, ensuring a more precise delineation of the canopy’s contour. The active contour model is a method used for image edge detection and contour optimization. It defines an energy function that guides the contour curve to move towards the target edges and ultimately converge to the true boundaries. The energy function *E* is typically composed of internal energy $E_{int}$ and external energy $E_{ext}$, as follows:

(3) $$Ε=E_{int}+E_{ext}$$

The internal energy $E_{int}$ is used to control the smoothness and elasticity of the curve, and is generally expressed as:

(4) $${Ε}_{int}=\alpha {\left|\frac{\partial C\left(s\right)}{\partial s}\right|}^{2}+\beta {\left|\frac{\partial^{2}C\left(s\right)}{\partial s^{2}}\right|}^{2}$$

where $C\left(s\right)$ is the contour curve, α and β are the weight coefficients controlling the smoothness and elasticity, and s is the arc-length parameter of the curve.

The external energy $E_{ext}$ is related to the image features, such as gradient information, and can be expressed as follows:

(5) $${Ε}_{ext}=-{\left|\nabla ΙC\left(s\right)\right|}^{2}$$

where Ι is the image grayscale value and ∇Ι is the gradient of the image.

By minimizing the energy function *E*, the optimal position of the contour curve can be obtained. This is typically solved using variational methods. By setting $\frac{\delta Ε}{\delta C}=0$, the evolution equation of the curve is derived. Through iterative solving of this equation, the contour progressively converges to the target boundary.

After obtaining the optimized contour mask image, the pixel mapping relationship between the mask and the true ruler was determined, facilitating the calculation of the actual cabbage canopy area. The mapping equation was derived from the pixel correspondence between the ruler image and the actual dimensions, enabling the conversion of image pixel areas to real physical areas.

Let the true length of the ruler be $L_{recog}$ (in units such as cm), and the corresponding pixel length in the image be $L_{pixel}$. The ruler factor k between pixels and actual length can be expressed as follows:

(6) $$k=\frac{L_{recog}}{L_{pixel}}$$

For any given region in the image with a pixel area $S_{pixel}$ (in terms of pixel count), the corresponding recognized physical area $S_{recog}$ is given by

(7) $$S_{recog}=k^{2}S_{pixel}$$

Since area is related to the square of length, the ruler factor must be squared.

**Figure 5:**
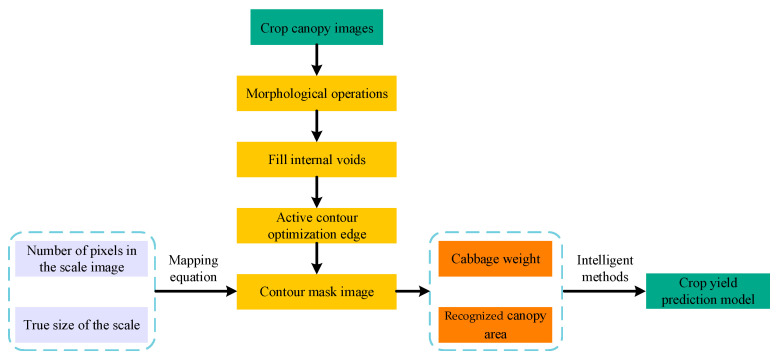
Design process of crop yield prediction model.

By correlating the recognized canopy area with cabbage weight, a predictive model for crop yield was further constructed using intelligent methods (Figure 5).

## 3. Results

### 3.1. Accuracy Analysis of CCPA Recognition

**Figure 6:**
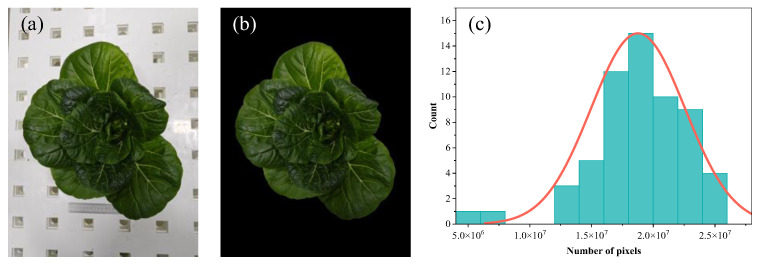
Pretreatment of crop canopy image. (**a**) Original image; (**b**) calibration image; (**c**) number of pixels in the projected area of the crop canopy recognition (normal distribution curve).

In the process of capturing crop canopy images, the original images, with a resolution of 6144 × 8192 pixels, are often influenced by background noise, which may undermine the accuracy of the subsequent analysis. A manual and meticulous background removal technique was employed to precisely exclude the background, allowing for the extraction of clear, unobstructed canopy images of each plant, which were subsequently used as calibration images. To guarantee the reliability of the calibration, an accurate pixel count of the CCPA was performed for each calibration image, offering a solid and quantifiable basis for the subsequent data processing and model development (Figure 6).

**Figure 7:**
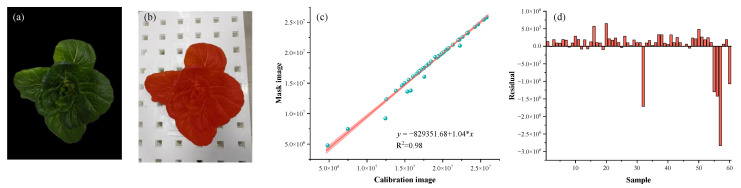
Linear analysis of calibration image and mask image. (**a**) Calibration image; (**b**) mask image; (**c**) linear fitting of the CCPA recognition; (**d**) residual histogram of the CCPA recognition.

In this research, crop color features, morphological operations, and optimization of the active contour model were employed to precisely identify the crop canopy contours. Corresponding mask images were then generated based on this identification. A regression analysis was conducted by comparing the pixel count of the mask image with the actual crop canopy projection area pixel count from a calibration image. The analysis revealed a strong linear correlation between the two, with an R^2^ value of 0.98, indicating the high accuracy of the CCPA recognition technique (Figure 7).

### 3.2. Real Calculation of CCPA Recognition

**Figure 8:**
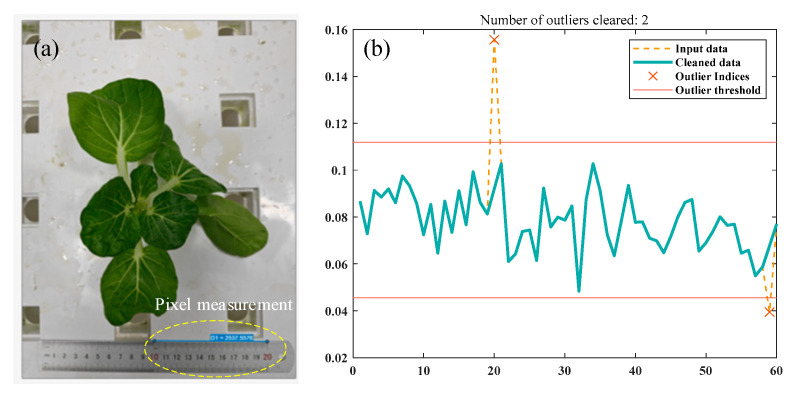
Image spatial resolution calculation. (**a**) Ruler scale pixel measurement; (**b**) outliers cleared.

In accordance with the scale of the reference ruler in the original image, a straight line was drawn on the image, precisely aligned with the ruler’s scale. The pixel count along this line was then recorded. To ensure the results’ representativeness and accuracy, multiple lines were drawn and measured at different positions along the ruler’s scale. Next, the pixel counts for all samples were analyzed, and outliers were cleared using the interquartile range (IQR) method. The average pixel count of the remaining samples was calculated, and this value, combined with the actual physical dimensions of the reference ruler, led to the determination of the spatial resolution of the images taken vertically at a height of 80 cm. The resulting spatial resolution was 0.078 mm/pixel. This spatial resolution reflects the actual physical area represented by each pixel in the image, ensuring the accuracy and reliability of the image analysis in this study (Figure 8).

Based on the calculation approach provided in Formula (7), the recognized area of the crop canopy outline was derived.

### 3.3. Construction of Crop Yield Prediction Model

In this study, 28 different modeling methods were chosen for crop yield prediction, encompassing multiple categories of models to ensure a thorough assessment of various prediction approaches. The model categories are summarized in Table 1.

**Figure 9:**
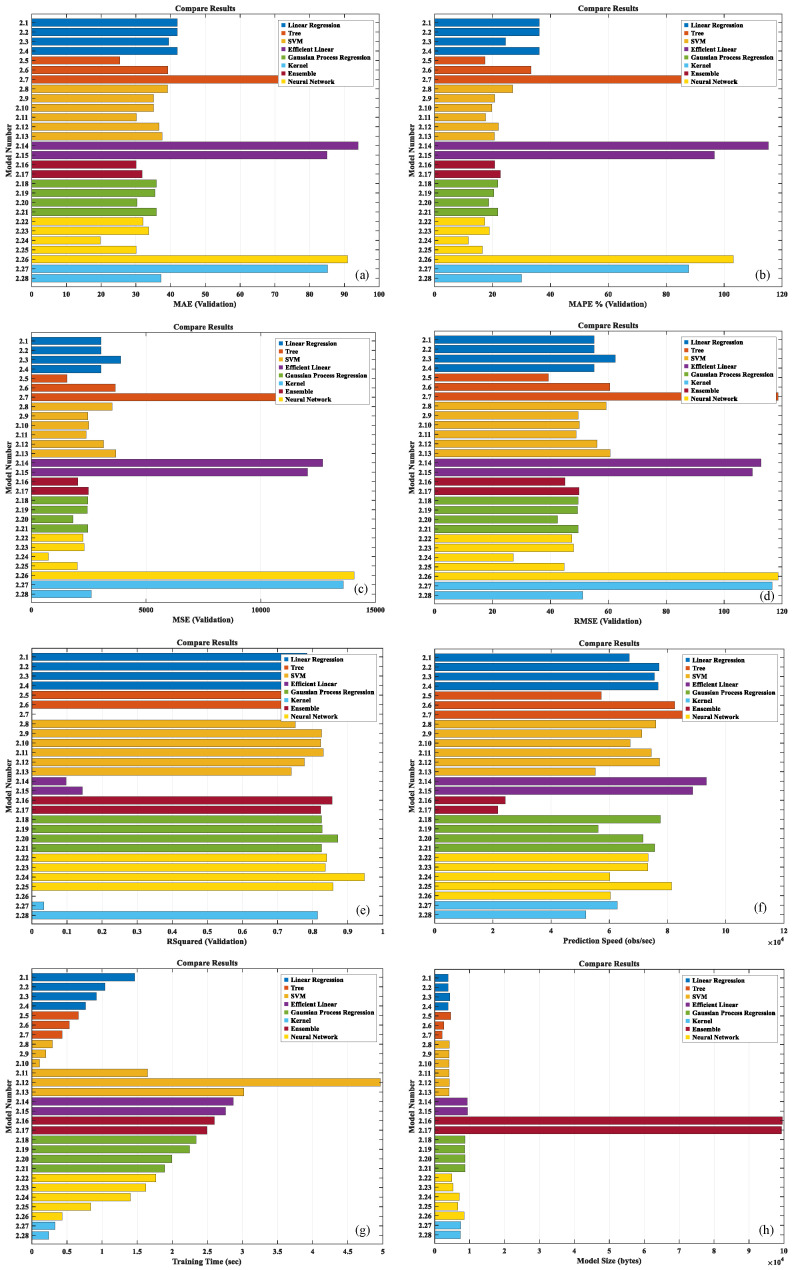
Model performance. (**a**) MAE; (**b**) MAPE; (**c**) MSE; (**d**) RMSE; (**e**) R^2^; (**f**) prediction speed; (**g**) training time; (**h**) model size.

After completing the training phase of the crop yield prediction models, we introduced an additional dataset to validate the models. This process aims to conduct a comprehensive assessment of the models’ predictive capabilities and stability, supported by a quantitative analysis using multiple performance metrics. The performance evaluation results for the models, which encompass several key indicators, including the mean absolute error (MAE), mean absolute percentage error root (MAPE), mean square error (MSE), mean square error (RMSE), coefficient of determination (R^2^), prediction speed, training time, and model size, are illustrated in Figure 9.

## 4. Discussion

### 4.1. Effect of Complex Background on CCPA Recognition

**Figure 10:**
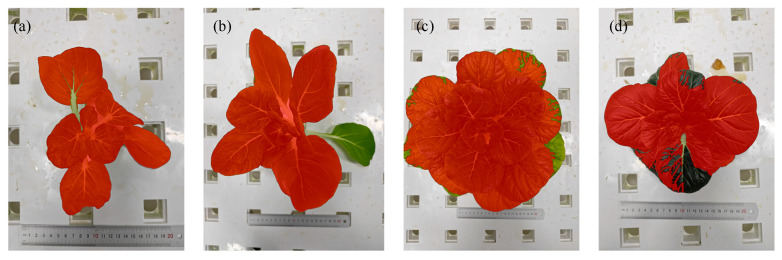
Mask images with larger errors. (**a**) Leaf stalk not recognized; (**b**) single leaf not recognized; (**c**) light-colored leaf not recognized; (**d**) dark-colored leaf not recognized.

Although there is a significant correlation between the number of pixels in the mask image and the number of pixels in the crop outline in the calibration image, a small number of samples still exhibit large errors in practical applications. Some of the mask images with larger errors are shown in Figure 10.

In Figure 10a, the color of the petiole is very similar to the white of the planting board, which presents a challenge for the algorithm to effectively differentiate between the petiole and the board’s boundary. This similarity leads to erroneous identification by the crop contour recognition algorithm [28]. In Figure 10b, the leaf’s posture is closely aligned with the surface of the planting board, and the instability of lighting during image capture creates shadows and highlights, further compromising the accuracy of the recognition algorithm [29]. Figure 10c,d show that the color of the leaf edges is either too dark or too light, exceeding the color threshold preset in the algorithm, resulting in the failure to correctly identify the leaf contour [30]. These issues highlight that the crop contour recognition algorithm in this study predominantly relies on color features, and its performance is limited when confronted with significant color variations.

Future research should explore the integration of multiple features, such as shape and texture, to enhance the robustness and accuracy of the algorithm under more complex environmental conditions.

### 4.2. Performance Analysis of Crop Yield Prediction Model

Model performance is a key indicator of its efficacy in addressing a specific task [31,32,33]. It represents the model’s ability to predict accurately when confronted with unseen data. The capacity to generalize effectively across diverse, unknown scenarios is a fundamental aspect of model evaluation, particularly in the context of real-world applications where data is often unpredictable [34]. In terms of MAE and MAPE metrics, the Wide Neural Network model (model number: 2.24) exhibits the lowest values, while the Efficient Linear Least Squares model (model number: 2.14) displays the highest values (Figure 9a,b). Regarding MSE and RMSE, the Wide Neural Network model (model number: 2.24) again shows the lowest values, with the Trilayered Neural Network model (model number: 2.26) presenting the highest values (Figure 9c,d). For the R^2^ metric, the Wide Neural Network model (model number: 2.24) achieves the highest value, while both the Coarse Tree model (model number: 2.7) and the Trilayered Neural Network model (model number: 2.26) attain a value of zero (Figure 9e). In terms of prediction speed, the Coarse Tree model (model number: 2.7) is the fastest, whereas the Bagged Trees model (model number: 2.17) is the slowest (Figure 9f). For training time, the Cubic SVM model (model number: 2.10) demonstrates the shortest training time, while the Medium Gaussian SVM model (model number: 2.12) exhibits the longest training time (Figure 9g). Regarding model size, the Coarse Tree model (model number: 2.7) is the smallest, and the Boosted Trees model (model number: 2.16) has the largest size (Figure 9h).

The top four models with the highest R^2^ values are selected from all the models: the Wide Neural Network model (model number: 2.24), the Fine Tree model (model number: 2.5), the Exponential GPR model (model number: 2.20), and the Bilayered Neural Network model (model number: 2.25). A summary of the eight metrics for these four models is presented in Table 2.

**Figure 11:**
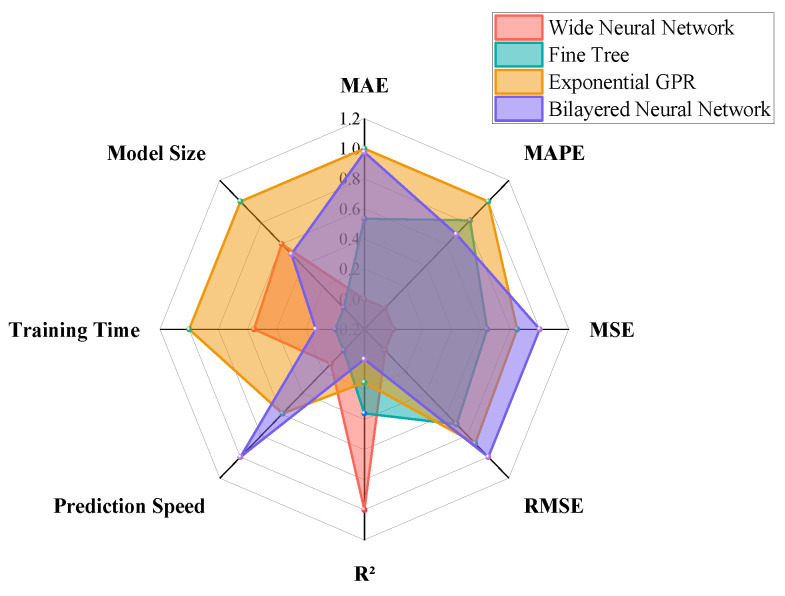
Comprehensive index radar chart.

To visually compare the performance of four different models across various performance metrics, this study uses eight key performance indicators to quantitatively evaluate each model. Considering that each indicator may have different dimensions and quantification methods, and to ensure the comparability of the indicators in the comparison, this study normalizes all the indicators, unifying their dimensions, so they can be effectively compared under the same standard (Figure 11).

**Figure 12:**
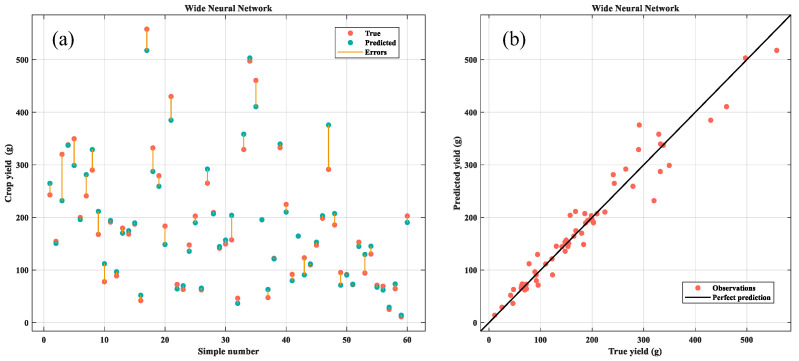
Crop yield prediction using wide neural networks. (**a**) Error condition; (**b**) linear fitting.

The Wide Neural Network model exhibits the smallest values in MAE, MAPE, and MSE, and achieves the highest R-squared values. In contrast, the Bilayered Neural Network model demonstrates the fastest prediction speed. Notably, model training Ttme is proportional to model size, with the Fine Tree model being the smallest in size and requiring the shortest training time. The Wide Neural Network model ranks third in both prediction speed and training time. When considering all evaluation metrics, the Wide Neural Network emerges as the most optimal method for crop yield prediction. This result underscores its superior predictive accuracy, robust pattern recognition capabilities, and stability across diverse scenarios (Figure 12).

## 5. Conclusions

This study addresses the challenges of crop yield prediction in plant factory environments by capturing crop canopy images and effectively eliminating background interference, thereby achieving accurate recognition of crop canopy contours. The coefficient of determination (R^2^) for the CCPA recognition reached 0.98, demonstrating the accuracy and stability of the canopy contour extraction. Based on this, by referencing the actual scale of a ruler and the corresponding pixel counts, and removing outliers through data processing, a spatial resolution of 0.078 mm/pixel was obtained.

To comprehensively assess the performance of prediction models, this study conducted a comparative analysis of 28 different models, evaluating them across multiple metrics, including MSE, RMSE, MAE, MAPE, R^2^, prediction speed, training time, and model size. Through optimization and selection, the Wide Neural Network model was chosen as the optimal solution for crop yield prediction. This model exhibited excellent predictive accuracy, with a predicted R^2^ of 0.95, an RMSE of 27.15 g, and a MAPE of 11.74%, indicating its high accuracy in crop yield forecasting. Furthermore, the model’s prediction speed reached 60,234.90 obs/sec, demonstrating strong real-time responsiveness. Additionally, the model size was 7039 bytes, making it suitable for rapid deployment and lightweight applications.

In the early seedling stage, crops are planted closely with small gaps between them. As the crops grow and reach a certain size, they are relocated to a cultivation room where they have more space to grow. The expanded space and reduced overlap between plants in the cultivation room allow the predictive model to capture the distinctive image features at various growth stages. As a result, the Wide Neural Network model effectively accommodates the changes throughout the growth process and provides real-time yield predictions for the entire growth period. From the seedling stage to maturity, it adjusts the predictions based on changes in the canopy size of the crops, ensuring both accuracy and real-time responsiveness. This all-encompassing predictive ability provides accurate decision-making insights for agricultural management, aiding in the optimization of planting strategies and improving the efficiency and precision of yield forecasting.

## Figures and Tables

**Table 1:** Model method for crop yield prediction.

Model Type	Model Name	Interpretability	Define Model Number
Linear Regression	Linear	Easy	2.1
	Interactions Linear	Easy	2.2
	Robust Linear	Easy	2.3
	Stepwise Linear	Easy	2.4
Tree	Fine Tree	Easy	2.5
	Medium Tree	Easy	2.6
	Coarse Tree	Easy	2.7
SVM	Linear SVM	Easy	2.8
	Quadratic SVM	Hard	2.9
	Cubic SVM	Hard	2.10
	Fine Gaussian SVM	Hard	2.11
	Medium Gaussian SVM	Hard	2.12
	Coarse Gaussian SVM	Hard	2.13
Efficient Linear	Efficient Linear Least Squares	Easy	2.14
	Efficient Linear SVM	Easy	2.15
Ensemble	Boosted Trees	Hard	2.16
	Bagged Trees	Hard	2.17
Gaussian Process Regression	Squared Exponential GPR	Hard	2.18
	Matern 5/2 GPR	Hard	2.19
	Exponential GPR	Hard	2.20
	Rational Quadratic	Hard	2.21
Neural Networks	Narrow Neural Network	Hard	2.22
	Medium Neural Network	Hard	2.23
	Wide Neural Network	Hard	2.24
	Bilayered Neural Network	Hard	2.25
	Trilayered Neural Network	Hard	2.26
Kernel	SVM Kernel	Hard	2.27
	Least Squares Kernel Regression	Hard	2.28

**Table 2:** Eight metrics for four models.

Metrics	Wide Neural Network	Fine Tree	Exponential GPR	Bilayered Neural Network
MAE	19.76	25.40	30.33	30.08
MAPE	11.74	17.45	18.67	16.54
MSE	737.33	1547.79	1808.31	2002.25
RMSE	27.15	39.34	42.52	44.75
R^2^	0.95	0.89	0.87	0.86
Prediction Speed (obs/sec)	60,234.9	57,224.6	71,582.0	81,433.2
Training Time (sec)	1.40	0.66	1.99	0.84
Model Size (bytes)	7039	4613	8643	6651

## Data Availability

Data are contained within the article.
